# Public’s calorie literacy and perceived effectiveness of restaurant menu calorie labeling in the United Arab Emirates

**DOI:** 10.1371/journal.pone.0293387

**Published:** 2023-10-24

**Authors:** Leila Cheikh Ismail, Hanin Kassem, Tareq M. Osaili, Mona Hashim, Reyad Obaid, Hadia Radwan, Maysm N. Mohamad, Sheima T. Saleh, Zein Al Zomut, Salma Abu Qiyas, Radhiya Al Rajaby, Rameez Al Daour, Habiba I. Ali, Lily Stojanovska, Ayesha S. Al Dhaheri

**Affiliations:** 1 Department of Clinical Nutrition and Dietetics, College of Health Sciences, University of Sharjah, Sharjah, UAE; 2 Nuffield Department of Women’s & Reproductive Health, University of Oxford, Oxford, United Kingdom; 3 Department of Nutrition and Food Technology, Faculty of Agriculture, Jordan University of Science and Technology, Irbid, Jordan; 4 Department of Nutrition and Health, College of Medicine and Health Sciences, United Arab Emirates University, Al Ain, UAE; 5 Jordanian Society for Food and Nutrition, Amman, Jordan; 6 Institute for Health and Sport, Victoria University, Melbourne, Australia; Tehran University of Medical Sciences, ISLAMIC REPUBLIC OF IRAN

## Abstract

Restaurant menu calorie labeling is regarded as a promising, cost-effective, and innovative method that will have an impact on the food environment, raise awareness among consumers, and aid in global efforts to prevent obesity. This study aimed to assess the public’s calorie literacy, dining practices, and perceived effectiveness of restaurant menu labeling implementation in the United Arab Emirates (UAE). A descriptive, web-based cross-sectional study was conducted among 1279 adults in the UAE. Socio-demographic characteristics, calorie literacy, and perceived effectiveness of restaurant menu labeling among participants were investigated. Chi-square analysis was used to compare indicators across demographic characteristics. More females than males completed the online survey, (56.1% and 43.9%). Most of the participants aged < 30 years old (54.8%), The majority of participants reported eating away from home at least once per week (89.6%). 66.0% of participants were knowledgeable about calorie definition, but only 37.1% knew about average daily energy requirements. Younger participants, with a normal BMI, and higher education levels reported a significantly higher likelihood of eating at a chain restaurant with caloric information on the menu (*p* <0.05). The majority of participants (76.0%) preferred to see calorie information and other nutrition information on menus. To conclude, menu labeling is a welcomed policy to be implemented in food outlets. Further investigation is necessary to ascertain the most efficacious method of presenting nutrition information to consumers to facilitate informed purchasing decisions considering the potential benefits of mandating calorie declaration in obesity prevention efforts.

## Introduction

The trend of consuming food away from home is on the rise globally [1]. This raises a lot of concern among health professionals as available evidence indicates its association with poor diet quality, nutritional status, and overall health [2]. Moreover, available data indicates that consumers often underestimate the energy content in their food, particularly food from fast-food restaurants. The consumption of such food is directly related to energy intake as food at restaurants is often served in larger portions leading to unintentional overconsumption [3, 4].

Global estimates of overweight and obesity are increasing, with more than 1.9 billion adults living with overweight, and over 650 million adults living with obesity in 2016 [5]. In the United Arab Emirates (UAE) in particular, the prevalence of overweight and obesity has at least doubled between 1989 and 2017 [6]. Several systematic reviews have shown that the region has been passing through a phase of nutrition transition, owing to factors such as population growth, urbanization, food globalization, and economic and cultural factors [7, 8]. More specifically, sedentary lifestyles and unfavorable dietary behaviors including increased consumption of energy-dense foods, larger portion sizes of food and beverages at chain restaurants, low intakes of fruits and vegetables, and generally poor food choices, are recognized as major contributors to the alarming surge in obesity rates in the region [9, 10].

Literature shows that people nowadays are consuming more calories than they need by substituting traditional foods with calorie-dense food options and consuming sugar-sweetened beverages (SSBs) which play a significant role in expanding obesity rates [11, 12]. In this sense, as global and local dietary trends shift towards increased consumption of food away from home, there is a crucial need for urgent innovative approaches at the individual, organizational, and retail levels that prevent obesity and other non-communicable diseases and promote healthy eating for populations.

The UAE provides a success story in establishing and implementing innovative enterprises such as the ‘Healthy Food School Box’ initiative which includes several innovative awareness activities that aim to promote food safety and healthy eating among children in the UAE [13], the ‘Mutabah system’ which screens school children and helps in promptly identifying those living with overweight and obesity [14], and the ‘Ma’kom’ initiative and the ‘Dubai Fitness Challenge’ that encourage people in the country to become more physically active [15, 16]. Moreover, in efforts to address the obesity epidemic, the UAE has levied an excise tax on energy drinks (100%) and carbonated drinks (50%) in 2019 [17]. The UAE along with many countries have also introduced or implemented nutritional labeling policies including front-of-pack labeling and nutrition facts panels among others [18, 19]. One of these is the recent “SEHHI” program to promote healthier food choices across the UAE’s capital city by placing a label on foods to label their healthiness launched by the Abu Dhabi Public Health Centre [20].

Historically, the scarcity of nutrition information in food retail establishments has been considered a factor contributing to the difficulty of making healthy food choices by consumers, suggesting the need for restaurant menu labeling [21]. Research confirms that consumers regardless of their age can make healthier food choices when the information is readily available to them [22]. To address this issue, in 2010, the Food and Drug Administration (FDA) in the United States required chain restaurants and other similar retail food establishments to declare the energy content of food items on their menus [23]. Promoting the use of nutrition information and enhancing calorie awareness among consumers have been found to have a positive impact on consumer behavior in choosing foods with lower-calorie options [24, 25]. To date, this policy has gained huge interest and support from individuals and health-concerned stakeholders as a potential strategy to combat the spread of obesity and was implemented in several countries globally on a mandatory basis including the United States [23], United Kingdom [26], and South Korea [27]. In the Arab region, restaurant menu labeling has been mandated in Saudi Arabia and implemented voluntarily in the UAE [28, 29].

Restaurant menu calorie labeling is regarded as a promising, cost-effective, and innovative method that will have an impact on the food environment, raise awareness among consumers, and aid in global efforts to prevent obesity [30]. Moreover, restaurant menu labeling supports better food choices among consumers and encourages food service establishments to reformulate their menus and include healthier options [30, 31]. Studies in the United States that investigated restaurant menu labeling effects on consumers’ food choices indicated a reduction of total calories ordered per consumer while using them ranging from 83 to 227 calories [32, 33]. Another study revealed that using these labels was associated with a moderately higher healthy eating index regardless of weight status [34]. Moreover, other studies indicated that consumers favor restaurant menu labeling and are interested in knowing the calorie count of the food they order [22, 35], while others found a minor impact on altering consumer behavior [36, 37]. Nonetheless, the literature suggests that restaurant menu labeling is a cost-effective method to support reducing obesity-related cancer burdens and lower healthcare costs [38, 39].

In line with the National Nutrition Strategy 2030 in the UAE and the alarming rates of obesity and overweight in the country, it is important to look into possible strategies that help mitigate the burden of these diseases [40]. Previous work by Radwan et al. investigated consumers’ attitudes toward the implementation of the calorie declaration policy in the UAE when the legislation draft was proposed [41]. The findings revealed that people in the UAE were in favor of mandating calorie labeling of restaurant menu items [41]. Since then, the first phase of the legislation was passed including the development of guiding specifications and voluntary implementation in many chain restaurants and other retail food outlets. Therefore, this study aimed to assess the public’s calorie literacy, dining practices, and perceived effectiveness of restaurant menu labeling after its voluntary implementation in the UAE.

## Methods

### Study design and participants

This is a cross-sectional web-based study conducted between July and October 2020 among adults in the UAE. The inclusion criteria were nationals and expatriates who were 18 years and older and living in the UAE. A convenient sample of a total of 1279 participants was drawn from all seven emirates in the UAE (Abu Dhabi, Dubai, Sharjah, and Northern Emirates; Ajman, Um Al Quwain, Ras Al-Khaimah, and Fujairah). A web link connecting to the online survey was shared on different social media platforms, e.g., LinkedIn™, Facebook™, and Instagram™. Participants were also recruited by sending invitation links via emails or WhatsApp™ to the researchers’ contacts. Snowball sampling was also used to ensure a wide range of participation by encouraging consenting participants to share the web link with their contacts. An information sheet explaining the objectives of the study, significance, and protocol was provided on the first page of the study followed by a consent form. An electronic informed consent was obtained from all participants. Only consenting participants were then directed to complete the questionnaire.

Participants were free to exit the survey at any point and data was collected anonymously. This study was conducted according to the guidelines in the Declaration of Helsinki. All procedures involving human subjects/patients were approved by the Research Ethics Committee at the University of Sharjah (Number: REC-20-06-25-04-S). The minimum required sample size was calculated based on the following equation with a confidence interval of 95%:

$$N=z^{2}\times P\times (1-P)/e^{2}$$

Where z = 1.96; P = (estimated proportion of the population that presents the characteristic) = 0.5; e (margin of error) = 0.05; N (sample size) = 384 participants, plus 20% (attrition rate) = approximately 461 participants.

### Survey questionnaire

The questionnaire was prepared on Google Forms in both English and Arabic languages. A first draft of the survey was developed by researchers in the field of nutrition and dietetics upon review of relevant literature by Bleich et al. [35] and Radwan et al. [41]. The questionnaire was subsequently modified to inquire about the demographics of participants, the perceived usefulness of calorie declaration, and dining practices. The survey was reviewed by a panel composed of three Ph.D. holders in the Clinical Nutrition and Dietetics Department and pilot-tested on 30 participants. The pilot testing data was not included in the results. The final questionnaire was translated from English to Arabic using the Brislin backtranslation method and the English version is provided in the supporting information section (S1 File).

The questionnaire included 21 close-ended questions and consisted of five parts. The first part included sociodemographic information such as sex, nationality, age group, marital status, employment status, household income level and education level. Self-reported weight and height were used to calculate the body mass index (BMI) by dividing the weight in kilograms by the height squared in meters (kg/m2). For categorization, the World Health Organization guideline for BMI classification was used [42]. The second part comprised two items: questions related to energy definition and knowledge of the recommended daily caloric requirement, while the third part included three items that inquired about consumer dining practices (i.e., frequency of eating away from home, preferred food outlets, and the factors that influence their purchasing decision). The fourth part included three items that addressed self-perceived caloric knowledge and views on the influence of calorie posting, and the fifth part covered participants’ perceptions of posting caloric information in chain restaurants. The last part also examined consumer preferences on calorie posting on menus, asking if they would like to see calories and other nutritional information posted on menus. The questionnaire took approximately 10 to 15 minutes to complete.

### Statistical analysis

Data was analyzed using Statistical Package for the Social Sciences (SPSS) ver. 26.0 (IBM, Chicago, IL, USA). Descriptive statistics for the sociodemographic characteristics were reported as counts and percentages. Mean responses and percentages of responses in each category were computed. Cross tabulations and chi-square tests were used to compare participants’ responses across demographic characteristics (age, educational level, family income). Results were significant for p-value < 0.05.

## Results

### Study sample characteristics

The sociodemographic characteristics of the study population are presented in Table 1. More females than males completed the online survey, (56.1% and 43.9% respectively). Most of the participants were young, aged < 30 years old (54.8%), single (52.9%), had a college degree or above (73.8%), and about a third had a monthly income of less than 10,000 AED (29.8%). Almost half of the participants were employed (47.1%). Most of the participants had normal weight (44.1%), while 34.5% and 16.8% of them were overweight or obese respectively.

**Table 1:** Sociodemographic characteristics of the study participants (n = 1279).

Characteristic	*n*	(%)
**Sex**		
Female	718	56.1
Male	561	43.9
**Age (years)**		
<30	701	54.8
≥30	578	45.2
**Emirate**		
Abu Dhabi	390	30.5
Dubai	227	17.7
Sharjah	334	26.1
Northern Emirates ^a^	328	25.6
**Marital status**		
Married	603	47.1
Single	676	52.9
**Educational level**		
Less than college	335	26.2
College or above	944	73.8
**Employment status**		
Unemployed ^b^	676	52.9
Employed	603	47.1
**Household income AED** ^**c**^		
Less than 10,000	381	29.8
10,000–20,000	200	15.6
20,000–30,000	145	11.3
More than 30,000	152	11.9
Prefer not to say	401	31.4
**Body Mass Index (kg/m** ^**2**^ **)**		
Underweight (<18.5)	59	4.6
Normal (18.5–24.9)	564	44.1
Overweight (25–29.9)	441	34.5
Obese (≥30)	215	16.8

^a^ Northern Emirates (Ajman, Umm AL Quwain, Ras Al Khaimah, Fujairah)

^b^ Unemployed include those who retired, students, and housewives.

^c^ AED, United Arab Emirates Dirhams.

### Knowledge of energy definition and recommended energy requirements

Participants’ knowledge of energy definition and daily energy requirements are shown in Table 2. Most of the participants correctly identified the term ‘calories in food’ as the amount of energy the food gives (66.0%), while a lesser proportion of them knew the correct average daily calorie requirement of 2000–2500 kcal (37.1%). Only 28.4% of the participants answered both questions correctly. Knowledge of correct energy definition differed significantly by sex and education level. Moreover, the participants’ estimation of the correct daily energy requirements for adults differed significantly by education level. Females (*p* = 0.019), and those with a college degree or above (*p*<0.001), had better knowledge of energy definition within each category, while only those with a higher education level had better knowledge of average daily requirement questions than others (*p*<0.001). Furthermore, those who had normal weight answered both questions correctly more than their counterparts (*p* = 0.029). No significant difference was observed in knowledge by age.

**Table 2:** The association of participants’ knowledge of energy definition and recommended energy requirements with sociodemographic variables and BMI using chi-square (n = 1279).

	Knowledge of energy definition	Knowledge of energy requirements	Total knowledge
	Answered correctly n (%)	Answered correctly n (%)	Answered both correctly n (%)
**Total**	844 (66.0)	475 (37.1)	363 (28.4)
**Sex**			
Female	454 (53.8)	264 (55.6)	197 (54.3)
Male	390 (46.2)	211 (44.4)	166 (47.7)
***P-value***	0.019	0.771	0.126
**Age (years)**			
<30	460 (54.5)	269 (56.6)	208 (57.3)
≥30	384 (45.5)	206 (43.4)	155 (42.7)
***P-value***	0.403	0.171	0.367
**Marital status**			
Married	400 (47.4)	221 (46.5)	164 (45.2)
Single	444 (52.6)	254 (53.5)	199 (54.8)
***P-value***	0.813	0.772	0.321
**Educational level**			
Less than college	196 (23.2)	96 (20.2)	71 (19.6)
College or above	648 (76.8)	379 (79.8)	292 (80.4)
***P-value***	0.001	<0.001	<0.001
**Employment**			
Employed	456 (54.0)	244 (51.4)	188 (51.8)
Unemployed	388 (46.0)	231 (48.6)	175 (48.2)
***P-value***	0.261	0.418	0.477
**Body Mass Index (kg/m** ^**2**^ **)**			
Underweight (<18.5)	30 (3.6)	16 (3.40)	11 (3.0)
Normal (18.5–24.9)	370 (43.8)	218 (45.9)	169 (46.6)
Overweight (25–29.9)	301 (35.7)	164 (34.5)	120 (33.1)
Obese (≥30)	143 (16.9)	77 (16.2)	63 (17.4)
***P-value***	0.069	0.354	0.029

*P*-values were calculated using chi-square tests among demographic categories.

### Dining practices

**Fig 1:**
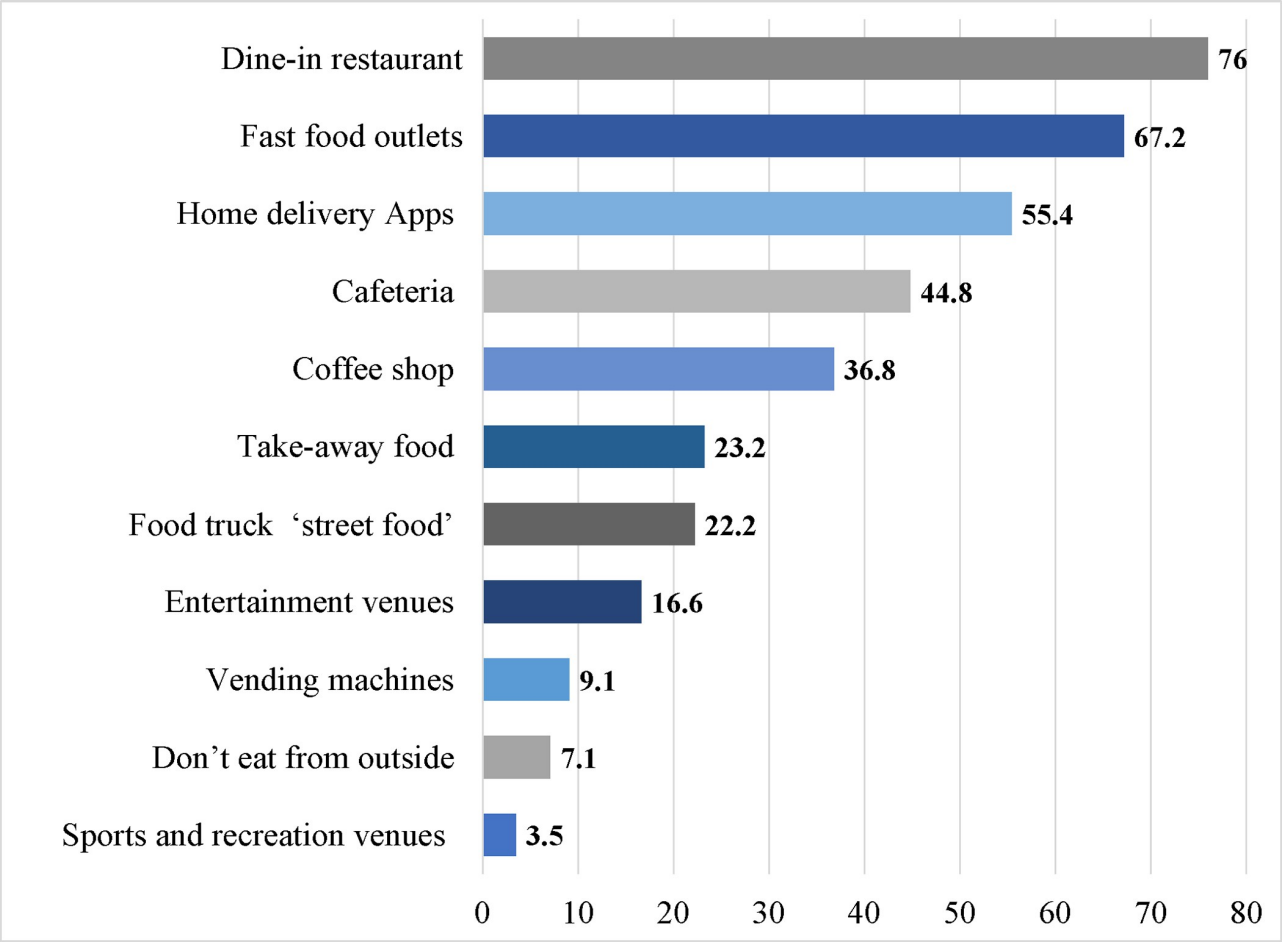
Participants’ distribution of preferred food outlets (n = 1279).

When asked about the frequency of eating away from home, most of the participants reported eating away from home one to two times per week (56.7%) as shown in Table 3. Moreover, when asked about the factors that influenced their food purchasing decisions, most participants reported mood and cravings followed by calorie content and price (62.2%, 19.4%, and 18.5% respectively). The most preferred food outlet options among participants were dine-in restaurants, followed by fast food outlets, and home-delivered food (76.0%, 67.2%, and 55.4% respectively) as shown in Fig 1.

**Table 3:** Percentage distribution of the participants’ dining practices (n = 1279).

Dining practices	n	(%)
**Frequency of eating away from home/week**		
None	133	10.4
1–2	725	56.7
3–4	288	22.5
5 or more	133	10.4
**Factors that influence purchasing food**		
Mood/Cravings	795	62.2
Calories and impact on health	248	19.4
Price of meal	236	18.5

### Self-perceived caloric knowledge and views on the influence of calorie posting in chain restaurants

The participants’ self-perceived caloric knowledge and the influence of calorie posting are presented in Table 4. About a third of the participants reported knowing enough about energy requirements to make better food choices (34.7%) and almost half of them reported not knowing enough but wanting to know more (46.9%). The chi-square test revealed that younger participants (<30 years), those who are single, who had a higher education level, who were employed and had a normal BMI perceived themselves as knowledgeable about energy requirements more significantly than their counterparts (*p*<0.001, *p*<0.001, *p* = 0.006, *p* = 0.003 and *p*<0.001 respectively). On the other hand, while older participants and those who are married answered negatively to this question, they expressed their preference in wanting to know more. Most participants agreed that when calorie count is provided on foods and beverages, it influences their food choices (78.1%). Older participants, those who were single, those who had a college degree, who were employed, and those with a normal BMI reported being more influenced by calorie posting in making their food choices than their counterparts (*p*<0.001).

**Table 4:** The association of sociodemographic variables with the public’s self-perceived caloric knowledge and views on the influence of calorie posting in chain restaurants using chi-square (n = 1279).

	Know enough about energy requirements to make lower-calorie choices			Calorie posting influences choosing lower-calorie meals	
	Yes	No, but want to know more	I don’t care	Yes	No
**Total**	444 (34.7)	60 (46.9)	235 (18.4)	999 (78.1)	280 (21.9)
**Sex**					
Female	244 (55.0)	350 (58.3)	124 (52.8)	571 (57.2)	147 (52.5)
Male	200 (45.0)	250 (41.7)	111 (47.2)	428 (42.8)	133 (47.5)
***P-value***	0.285			0.094	
**Age (years)**					
<30	243 (54.7)	295 (42.1)	163 (69.4)	509 (51.0)	192 (68.6)
≥30	201 (45.3)	305 (50.8)	72 (30.6)	40 (49.0)	88 (31.4)
***P-value***	<0.001			<0.001	
**Marital status**					
Married	214 (48.2)	309 (51.5)	80 (34.0)	512 (51.3)	91 (32.5)
Single	230 (51.8)	291 (48.5)	155 (66.0)	487 (48.7)	189 (67.5)
***P-value***	<0.001			<0.001	
**Educational level**					
Less than college	96 (21.6)	162 (27.0)	77 (32.8)	230 (23.0)	105 (37.5)
College or above	348 (78.4)	438 (73.0)	158 (67.2)	769 (77.0)	175 (62.5)
***P-value***	0.006			<0.001	
**Employment**					
Employed	238 (53.6)	337 (56.2)	101 (43.0)	557 (55.8)	119 (42.5)
Unemployed	206 (46.4)	263 (43.8)	134 (57.0)	442 (44.2)	161 (57.5)
***P-value***	0.003			<0.001	
**Body Mass Index (kg/m** ^**2**^ **)**					
Underweight (<18.5)	16 (3.6)	22 (3.7)	21 (8.9)	28 (2.8)	31 (11.1)
Normal (18.5–24.9)	188 (42.3)	251 (41.8)	125 (53.2)	419 (41.9)	145 (51.8)
Overweight (25–29.9)	157 (35.4)	220 (36.7)	64 (27.2)	366 (36.6)	75 (26.8)
Obese (≥30)	83 (18.7)	107 (17.8)	25 (10.6)	186 (18.6)	29 (10.4)
***P-value***	<0.001			<0.001	

*P*-values were calculated using a chi-square test among demographic categories.

Table 5 shows the participants’ views on calorie posting in chain restaurants. Around a third of the participants reported that they would more likely eat at chain restaurants with calorie information on menus (34.9%) and half of them trust that the calorie information on menus is correct (50.1%). Participants’ perceptions of calorie posting differed significantly between different sociodemographic characteristics. Younger participants, married, those with a higher education level, and those with a normal BMI reported a significantly higher likelihood than their counterparts of eating at a chain restaurant with caloric information on the menu (*p* = 0.001, *p* = 0.002, *p*<0.001 and *p* = 0.001 respectively). Moreover, those with a normal BMI were significantly more trusting of the correctness of calorie information on menus (*p* = 0.014).

**Table 5:** The association of sociodemographic variables with participants’ perceptions of posting caloric information in chain restaurants using chi-square (n = 1279).

	Likelihood of eating at chain restaurants with calorie information on the menu			Trust that the calorie information on menus is correct	
	More likely	Less likely	Neutral	Yes	No
**Total**	446 (34.9)	182 (14.2)	651 (50.9)	641 (50.1)	638 (49.9)
**Sex**					
Female	249 (55.8)	106 (58.2)	363 (55.8)	372 (58.0)	346 (54.2)
Male	197 (44.2)	76 (41.8)	288 (44.2)	269 (42.0)	292 (45.8)
***P-value***	0.826			0.094	
**Age (years)**					
<30	232 (52.0)	87 (47.8)	382 (58.7)	335 (52.3)	366 (57.4)
≥30	214 (48.0)	95 (52.2)	269 (41.3)	306 (47.7)	272 (42.6)
***P-value***	0.011			0.067	
**Marital status**					
Married	227 (50.9)	100 (54.9)	276 (42.4)	319 (49.8)	284 (44.5)
Single	219 (49.1)	82 (45.1)	375 (57.6)	322 (50.2)	354 (55.5)
***P-value***	0.002			0.060	
**Educational level**					
Less than college	81 (18.2)	55 (30.2)	199 (30.6)	158 (24.6)	177 (27.7)
College or above	365 (81.8)	127 (69.8)	452 (69.4)	483 (75.4)	461 (72.3)
***P-value***	<0.001			0.208	
**Employment**					
Employed	239 (53.6)	106 (58.2)	331 (50.8)	341 (53.2)	335 (52.5)
Unemployed	207 (46.4)	76 (41.8)	320 (49.2)	300 (46.8)	303 (47.5)
***P-value***	0.195			0.805	
**Body Mass Index (kg/m** ^**2**^ **)**					
Underweight (<18.5)	12 (2.7)	4 (2.2)	43 (6.6)	22 (3.4)	37 (5.8)
Normal (18.5–24.9)	178 (39.9)	82 (45.1)	304 (46.7)	264 (41.2)	300 (47.0)
Overweight (25–29.9)	167 (37.4)	62 (34.1)	212 (32.6)	238 (37.1)	203 (31.8)
Obese (≥30)	89 (20.0)	34 (18.7)	92 (14.1)	117 (18.3)	98 (15.4)
***P-value***	0.001			0.014	

*P*-values were calculated using a chi-square test among demographic categories.

**Fig 2:**
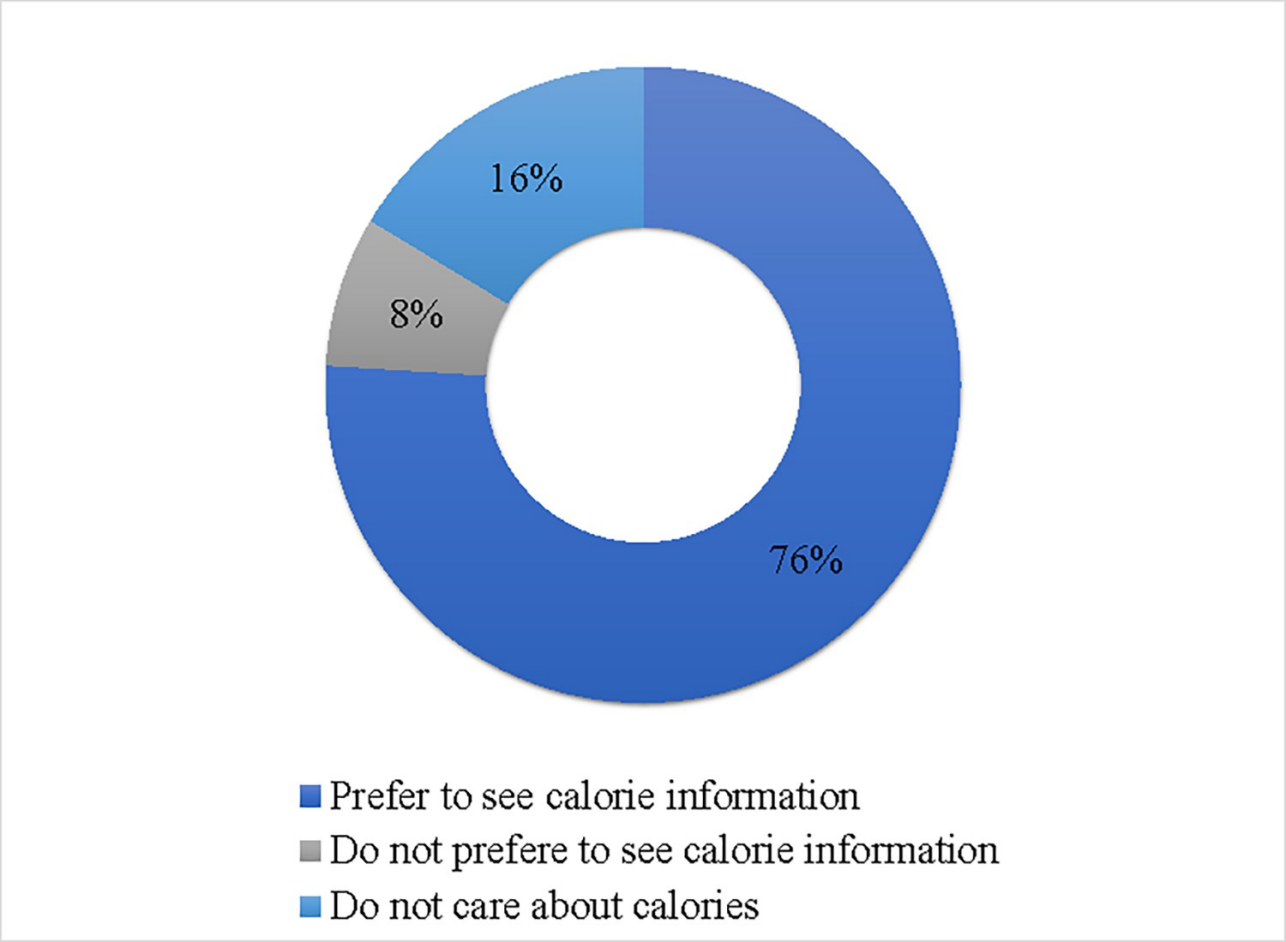
Participants distribution of preference to see calorie information on menus (n = 1279).

Most of the participants favored menu labeling and reported a preference for viewing caloric information on menus (76.0%) as shown in Fig 2.

**Fig 3:**
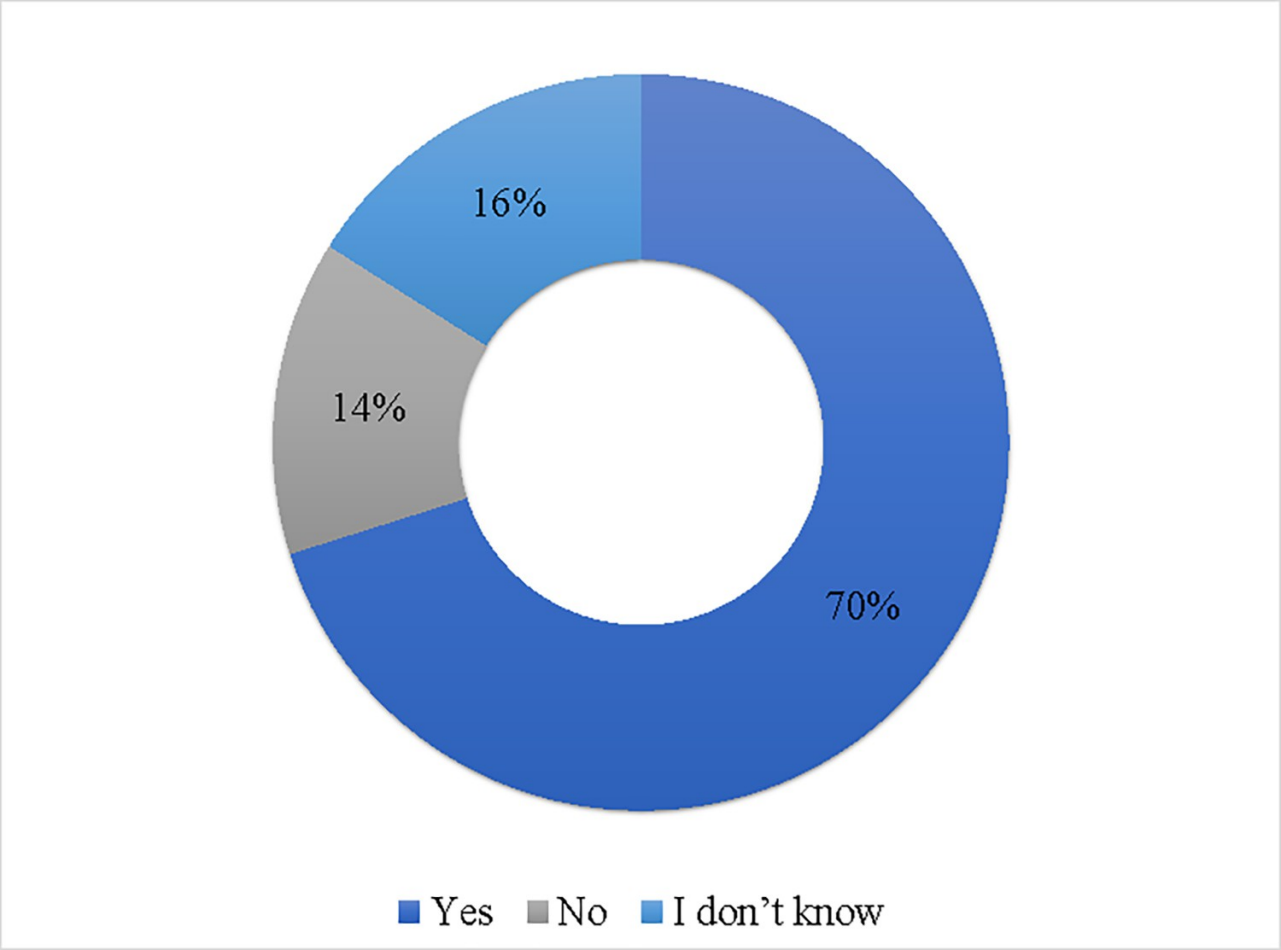
Participants distribution of preference to see other nutrition information on menus (n = 1279).

**Fig 4:**
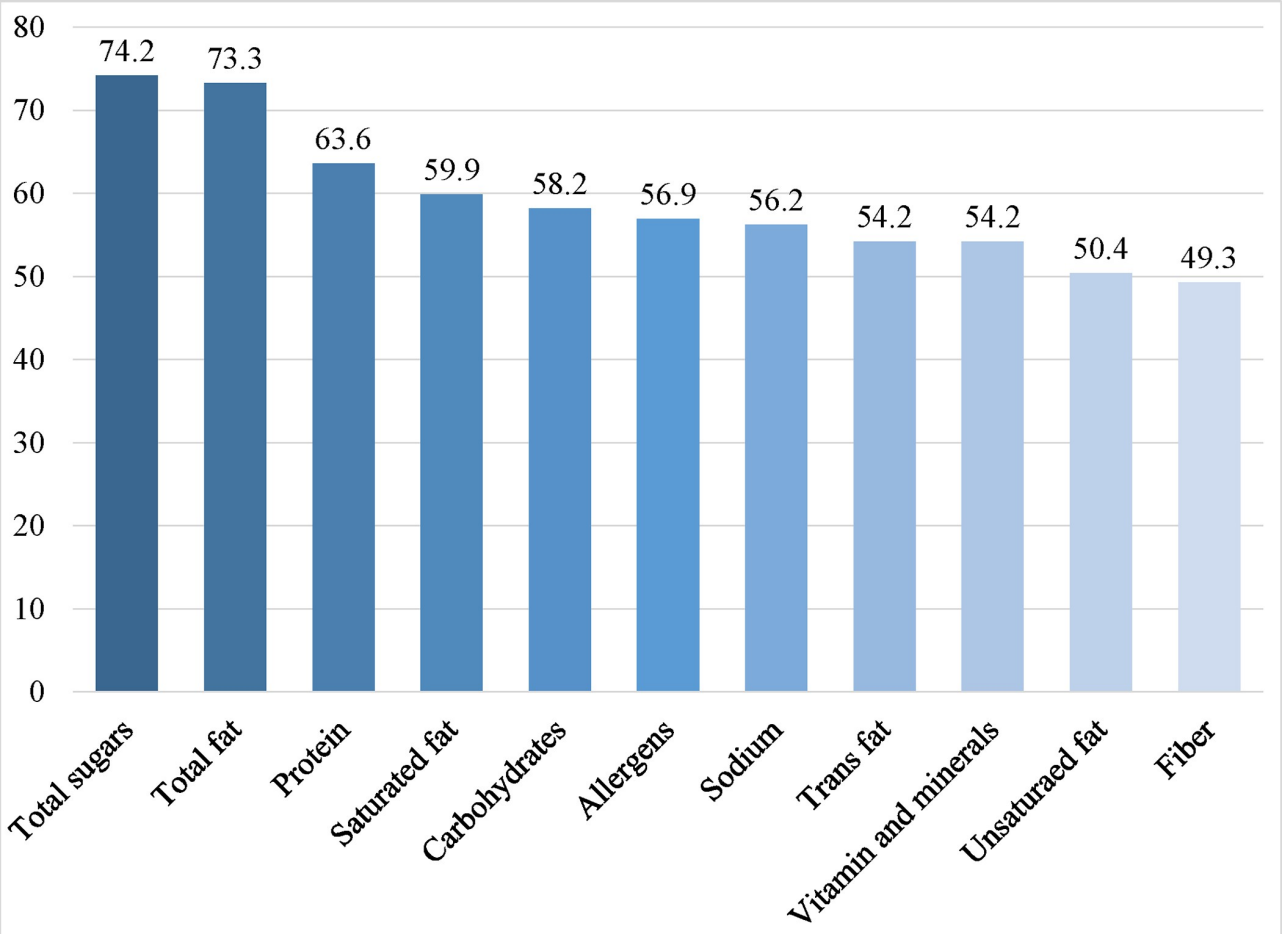
Other nutritional information participants would like to see on menus (n = 1279).

Over two-thirds of the participants (70.0%) reported a preference for viewing nutritional information on menus besides the calorie content as shown in Fig 3. When asked about which other nutritional information participants would like to see on menus, total sugars, total fat, and protein were the most common choices (>60%). Other information included saturated fats, carbohydrates, allergens, and sodium (Fig 4).

## Discussion

This is the first study in the UAE to evaluate the public’s perceived effectiveness towards the voluntary restaurant menu calorie labeling policy (introduced in 2020) in food establishments around the UAE. This study highlighted several knowledge gaps among the UAE participants concerning energy requirements, dining practices, and beliefs about the influence of posting caloric information on menus in different food outlets.

Overall, most participants were aware of energy definition but lacked knowledge of the average daily energy requirements. The findings of the present study differed from similar studies in the UAE, Saudi Arabia, and the United States, where most participants correctly identified the recommended daily energy requirements [35, 41, 43]. Previous studies in the UAE have highlighted a gap in nutritional knowledge and urged the need for policies to address nutrition literacy in the country among different age groups [44, 45].

In the present study, only a third of participants perceived themselves as knowledgeable about energy requirements enough to make lower-calorie food choices in restaurants (35%) which was lower than the proportion of participants reporting that in the United States (54%) [35]. Although about half of the participants reported not knowing enough about energy requirements to make lower-calorie food choices, they were interested in knowing more. This emphasizes the crucial need for educating the public on the assessment of energy requirements for food and nutrition and choosing healthier food more efficiently and indicates the willingness and potentially the readiness of consumers for such interventions.

Literature indicates that food away from home is generally less healthy and is significantly associated with a higher prevalence of obesity [11, 46]. In the present study, most participants reported eating away from home at least once a week which is similar to findings reported previously in the UAE, the United States, and Vietnam [35, 41, 47]. Moreover, similar to the study among Vietnamese consumers, dine-in and fast-food restaurants were the most frequently visited food outlets by our participants [47]. Such results emphasize the importance of providing calorie information to consumers in chain restaurants and different food outlets so that they are better informed about the energy content and thus make informed choices. Mood and cravings were chosen by most of the participants as factors affecting food choice highlighting the strong link between eating behavior and emotion [48]. A recent meta-analysis revealed an inverse relationship between emotional intelligence and disordered eating [49], suggesting how identifying emotional intelligence can help in identifying people at risk of disordered eating. From another perspective, more emphasis should be put toward educating the public about interoceptive awareness which refers to being aware of bodily signals of hunger and satiety [50, 51]. In the present study, the calorie content of food ranked second in factors that affect purchasing food away from home, followed by price. This was similar to the findings among Vietnamese consumers [47], however, it differed from those in the United States, where participants were evenly distributed between calories and price as influencing factors in food purchasing [35] suggesting the important impact for menu declaration.

The results of the present study have also indicated that participants have positive attitudes toward calorie posting on menus, as most of them reported that it influences their food choices and that they trust the information provided. Similar findings were observed among consumers in Saudi Arabia and the United States as most participants favored calorie posting and thought that it was useful [35, 52]. Public positive attitude and support for menu labeling and nutrition labeling in general, are key factors in the successful implementation of such policies. About a third of participants in our study indicated that they were more likely to eat at restaurants that provide calorie posting on menus. These findings were lower than those in Saudi Arabia [43] and higher than those in the United States and the UAE (prior to the voluntary implementation of menu labeling) [35, 41]. These findings suggest the need for more in-depth studies investigating barriers to menu labeling, particularly when reports show that more than 1,000 food outlets in the Emirate of Dubai alone have been displaying menu labeling since 2020 [53] and Abu Dhabi, the UAE’s capital are working on the “SEHHI for Displaying Calories on the Menu” as part of the SEHHI initiative [20].

The present study indicates that there were some differences in knowledge and perceptions of calorie posting between different socio-demographic characteristics. Participants who had a college degree or above, and those with normal BMI had better knowledge scores and generally more positive attitudes toward calorie posting. These findings are similar to those reported by previous studies conducted in the UAE and among Vietnamese consumers [41, 47]. Well-educated people are more likely to have healthy behaviors and pursue health information more regularly [54]. Moreover, participants with normal BMI had more positive perceptions toward calorie posting than their counterparts. A possible explanation would be that normal-weight people could be more aware and interested in seeking healthy options to maintain their weight status and overall health. In addition, females were more knowledgeable about energy definition, similar to other studies, as women generally are more interested in menu labeling to help them choose lower-calorie options and control their calorie intake [35, 41, 47].

Favorably, our analyses indicate that participants were interested in calorie posting and would like to see calorie information on menus and expressed a preference in seeing other nutritional detailed information. This was in line with similar studies indicating that consumers favor menu labeling and would like to have sufficient information to choose healthier food options [22, 35, 41, 47]. Two studies showed the positive impact of calorie posting on consumers’ purchasing decisions, as they recorded a 6% drop in the average calories per transaction at a common coffee shop [55] and a 14% reduction in calorie consumption in people who were provided with calorie labels [22]. Although a limited number of studies measured the positive impact of calorie posting on consumers’ choices, literature shows that some companies may be reformulating their products in line with the menu labeling requirements, by modifying portion sizes or ingredients [56, 57]. This implies that restaurant menu labeling may influence both consumers and food retailers. Furthermore, most participants in our study expressed their preference for viewing more calorie information posted in different food outlets, confirming that individuals are interested in menu labeling and would like to see it in food outlets they visit frequently.

Restaurant menu labeling is a new policy implemented voluntarily and has not been implemented widely in the UAE. As such, the consumers may not be well exposed to this concept yet, which warrants further research to be conducted in exploring its acceptance. It should be noted that this study has several limitations. The use of an online self-administered questionnaire and the nature of the cross-sectional design used may have led to a less representative sample which may have affected the external validity of the results. However, multiple methods of participant recruitment and through different channels were employed to minimize risks of self-selection bias and to capture a larger diversity of participants. Moreover, the methodology used does not give a clear representation of real-life purchasing behavior and does not measure the direct impact of menu labeling on consumer behavior. Despite this, this study has several strengths. It identified gaps in actual knowledge and consumer-perceived knowledge. Also, it highlighted the possible impact of a wider and mandatory menu labeling implementation on consumer practices and gave us a snapshot of consumers’ dining practices in the UAE and their future preferences concerning menu labeling and calorie information.

## Conclusions

Our findings propose that menu labeling and providing nutrition information are welcomed policies to be implemented widely in most food outlets since the majority of the participants exhibited positive attitudes and preferences. Given the surge of restaurants and other food outlets in the UAE, and with increasing consumers’ tendency for food away from home, mandating calorie declaration would be a useful policy in supporting obesity prevention strategies and enhancing consumers purchasing decisions. As such it is necessary to introduce more efficient educational interventions to the public on the importance of healthy eating as well as proper reading understanding and interpreting different food labels. More research is required to determine the optimum approach and best presentation to deliver nutrition information to consumers to help them make informed choices. Moreover, future studies are required to fill in knowledge gaps and provide clearer insights into the true impact of menu labeling on consumers and food outlets in the UAE.

## Supporting information

S1 File Survey questionnaire. (PDF)
