# Supplementary material for: Public’s calorie literacy and perceived effectiveness of restaurant menu calorie labeling in the United Arab Emirates
Source: PLoS One. 2023 Oct 24;18(10):e0293387. doi: 10.1371/journal.pone.0293387 (PMC10597515; doi:10.1371/journal.pone.0293387)
Supplement: S1 File — (PDF) [file pone.0293387.s001.pdf]

Demographics

1. **What is your gender?**
   - ☐ Female
   - ☐ Male
2. **What is your nationality?**
   - ☐ Local citizen
   - ☐ Resident Expatriate
   - ☐ Non-resident
3. **Which Emirate do you reside in?**
   - ☐ Abu Dhabi
   - ☐ Dubai
   - ☐ Sharjah
   - ☐ Northern Emirates (Ajman, Umm AL Quwain, Ras Al Khaimah, Fujairah)
4. **Which age group do you belong to?**
   - ☐ 18-30
   - ☐ 31-45
   - ☐ 46-65
   - ☐ 65+
5. **What is your marital status?**
   - ☐ Married
   - ☐ Single
   - ☐ Other
6. **What is your employment status?**
   - ☐ Employed
   - ☐ Retired
   - ☐ Other (e.g., homemaker, student)
7. **What is your household income?**
   - ☐ Less than 10,000 AED/MONTH
   - ☐ 10,000 to 20,000 AED/MONTH
   - ☐ 20,000 to 30,000 AED/MONTH
   - ☐ MORE THAN 30,000 AED/MONTH
   - ☐ I prefer not to tell
8. **What is your education Level?**
   - ☐ Less than high school
   - ☐ High school diploma
   - ☐ More than high school
9. **What is your weight? __________(kg)**
10. **What is your height? ___________ (cm)**

Recommended energy requirements and calorie knowledge

11. What does the term "Calories in food" mean to you?

- ☐ The amount of energy the food gives
- ☐ The amount of energy I burn when I eat this food
- ☐ It is related to fat
- ☐ I don't know

12. What is the average daily caloric requirement for adults?

- ☐ <1500 kcal
- ☐ 1500 – 1900 kcal
- ☐ 2000 - 2500 kcal
- ☐ > 2700 kcal
- ☐ I do not know

Consumer dining practice

13. How many times a week do you eat outside?

- ☐ None
- ☐ 1-2
- ☐ 3-4
- ☐ 5+

14. Do you get meals from the following food outlets? (Tick all that apply)

- ☐ Fast food
- ☐ Coffee shop
- ☐ Dine-in restaurant
- ☐ Cafeteria
- ☐ take-away from supermarket store
- ☐ Food truck / food stand / 'street food'
- ☐ Sports and recreation venues (Eg – stadium)
- ☐ Entertainment venue (eg – Theatres)
- ☐ Delivery Apps (Like Zomato, Talabat etc.)
- ☐ Vending machines
- ☐ I don't eat from outside

15. Which of the following factors influence your purchase at food outlets the most?

- ☐ Calories and their impact over health
- ☐ Price of meal
- ☐ Mood/Cravings

Self-perceived caloric knowledge and Usefulness of calorie posting

16. Do you feel that you know enough about your daily calorie requirements to make healthier food choices in restaurants?

- ☐ Yes, I know enough
- ☐ No, I do not know enough, but I would like to know more.
- ☐ It doesn't matter to me

17. Would the number of calories in foods and drinks influence your choice?

- ☐ Yes
- ☐ No

Perceptions of posting caloric information in chain restaurants

18. Would you be more or less likely to eat at restaurants if they reported calorie information on menus/menu boards/online for each food item? *

- ☐ More likely
- ☐ Less likely
- ☐ Neutral

19. Would you trust that the calorie information on menus is correct?

- ☐ Yes
- ☐ No
- ☐ I don’t know

Consumer preferences on calorie posting on menus

20. Would you like to see calorie information on menus?

- ☐ Yes
- ☐ No
- ☐ I don’t care about calories

21. In addition to calories, would you like to see other nutritional information on menus? *

- ☐ Yes
- ☐ No
- ☐ I don't know

22. if Yes, please tick the nutritional information you would like to see on menus. *

- ☐ Total Sugars
- ☐ Total fat
- ☐ Saturated fat
- ☐ Unsaturated fat
- ☐ Trans fat
- ☐ Sodium
- ☐ Dietary Fibers
- ☐ Carbohydrates
- ☐ Protein
- ☐ Vitamins/Minerals
- ☐ Allergen information
